# Retrograde intubation: An alternative in difficult airway management in the absence of a fiberoptic laryngoscope

**DOI:** 10.4103/0019-5049.72662

**Published:** 2010

**Authors:** Kishan Rao Bagam, SGK Murthy, C Vikramaditya, V Jagadeesh

**Affiliations:** Department of Anaesthesiology and Critical Care, Mamata Medical College, Khammam, Andhra Pradesh, India

Sir,

A 60-year-old male patient weighing 82 kg was posted for emergency evacuation of a subdural haematoma. Pre-anaesthetic evaluation of the patient revealed no abnormalities, with the patient being semiconscious, GCS 10 and showing no neurological deficit. On examination of the airway, all parameters such as mouth opening, lip biting, Tempero mandibular joint subluxation and thyromental and mentohyoid distance were within normal limits and were Mallampati grade II.

The patient was pre-medicated with 1 mg of Midazolam, 0.2 mg of Glycopyrrolate and 100 *μ*g of Fentanyl intravenously (IV). After induction with thiopentone sodium 300 mg IV, 6 mg of Vecuronium bromide IV was given and it was noted that although mask ventilation was possible, there was some resistance to air entry into the trachea, as suggested by reservoir bag resistance and inflation of the stomach. To relive the airway resistance, Guedel’s airway was inserted. After insertion of airway, airway resistance was decreased and saturation was maintained. On attempting endotracheal intubation, the epiglottis could be visualized but the glottis could not be visualized even after external laryngeal pressure was applied. The third time, we attempted the gum elastic bougie for intubation of the patient, with the epiglottis as a guide. When this attempt also failed, we put a call to the ENT surgeon to establish a surgical airway. While waiting for the surgeon, because the patient was already anaesthetized, we could attempt an emergency retrograde intubation.

Cricothyriod membrane puncture was performed with a 16 G needle and a guide wire used in central venous cannulation was advanced cephalad through the needle, the larynx and out of the mouth; the tracheal tube was passed over the guide wire. We went ahead with the procedure and timed ourselves till we successfully observed equal bilateral air entry after endotracheal intubation. The entire procedure took only 145 s. The surgical procedure and anaesthesia were uneventful. On completion, the residual neuromuscular blockade was reversed and the patient was shifted to the Neuro-ICU and extubated after full recovery of consciousness.

This technique was first reported by D.J. Waters in 1963.[1] The use of retrograde wire technique to assist in the management of difficult airway was first reported in 1981. Since then, modifications of this technique have included the use of the fiberoptic bronchoscope to permit tracheal intubation under direct visual control.[2] Difficult intubation is defined as inadequate visualization of the glottis and failed tracheal intubation as inability to insert a tracheal tube from the oropharynx into the trachea.[3] This technique may be useful in trauma patients requiring cervical spine immobilization as well as in patients with facial trauma, trismus, ankylosis of the jaw and cervical spine, upper airway masses and bleeding.

Fiberoptic intubation, a more recent technology, but technically demanding, is considered the safest and the most-effective method in known or suspected cases of difficult intubation. Its primary advantage is that it permits direct visual control of the intubation procedures.[4] However, in developing countries, the flexible fiberoptic laryngoscope usage is rare and, even when present, it requires expertise to use. Bleeding in the oropharynx can obscure the airway with a fiberoptic laryngoscope, even for an experienced anaesthetist. Alternative methods may be needed.[5] Retrograde intubation is the better alternative airway management in difficult airway conditions where there is no fiberoptic laryngoscope.
